# Exploring Associations Between the Self-Reported Values, Well-Being, and Health Behaviors of Finnish Citizens: Cross-Sectional Analysis of More Than 100,000 Web-Survey Responses

**DOI:** 10.2196/12170

**Published:** 2019-04-22

**Authors:** Anita Marianne Honka, Elina Helander, Misha Pavel, Holly Jimison, Pekka Mustonen, Ilkka Korhonen, Miikka Ermes

**Affiliations:** 1 Smart Health VTT Technical Research Centre of Finland Ltd Tampere Finland; 2 Faculty of Medicine and Health Technology Tampere University Tampere Finland; 3 College of Computer and Information Science Northeastern University Boston, MA United States; 4 Bouvé College of Health Sciences Northeastern University Boston, MA United States; 5 Duodecim Medical Publications Ltd Helsinki Finland

**Keywords:** value orientation, happiness, health behavior, healthy lifestyle, cross-sectional survey

## Abstract

**Background:**

Understanding the relationship between personal values, well-being, and health-related behavior could facilitate the development of engaging, effective digital interventions for promoting well-being and the healthy lifestyles of citizens. Although the associations between well-being and values have been quite extensively studied, the knowledge about the relationship between health behaviors and values is less comprehensive.

**Objective:**

The aim of this study was to assess retrospectively the associations between self-reported values and commitment to values combined with self-reported well-being and health behaviors from a large cross-sectional dataset.

**Methods:**

We analyzed 101,130 anonymous responses (mean age 44.78 years [SD 13.82]; 78.88%, 79,770/101,130 women) to a Finnish Web survey, which were collected as part of a national health promotion campaign. The data regarding personal values were unstructured, and the self-reported value items were classified into value types based on the Schwartz value theory and by applying principal component analysis. Logistic and multiple linear regression were used to explore the associations of value types and commitment to values with well-being factors (happiness, communal social activity, work, and family-related distress) and health behaviors (exercise, eating, smoking, alcohol consumption, and sleep).

**Results:**

Commitment to personal values was positively related to happiness (part *r*^*2*^=0.28), communal social activity (part *r*^*2*^=0.09), and regular exercise (part *r*^*2*^=0.06; *P*<.001 for all). Health, Power (social status and dominance), and Mental balance (self-acceptance) values had the most extensive associations with health behaviors. Regular exercise, healthy eating, and nonsmoking increased the odds of valuing Health by 71.7%, 26.8%, and 40.0%, respectively (*P*<.001 for all). Smoking, unhealthy eating, irregular exercise, and increased alcohol consumption increased the odds of reporting Power values by 27.80%, 27.78%, 24.66%, and 17.35%, respectively (*P*<.001 for all). Smoking, unhealthy eating, and irregular exercise increased the odds of reporting Mental balance values by 20.79%, 16.67%, and 15.37%, respectively (*P*<.001 for all). In addition, lower happiness levels increased the odds of reporting Mental balance and Power values by 24.12% and 20.69%, respectively (*P*<.001 for all).

**Conclusions:**

The findings suggest that commitment to values is positively associated with happiness and highlight various, also previously unexplored, associations between values and health behaviors.

## Introduction

### Background

Suboptimal health behaviors are significant determinants of poor health outcomes. However, the adoption of healthy lifestyles has not been sufficient at the population level, and obesity levels are increasing worldwide. In addition, the burden of mental health problems is growing [1,2]. Personal electronic health (eHealth) and mobile health (mHealth) interventions have great potential in empowering individuals to take care of their health and well-being in a cost-effective way [3,4]. However, the problem of low user engagement commonly prevents these interventions from achieving their full potential [4,5].

Various computer-tailored eHealth interventions have demonstrated that personalizing the content to the characteristics of individual users tend to be efficacious for promoting healthy behaviors [4,6,7], though engaging the unmotivated proportion of the population, not actively interested in their health, is always challenging [8]. The common tailoring variables found in eHealth or mHealth interventions are health behaviors and the readiness to change behavior [9,10], and some have also considered demographics, clinical risk factors, and personal information needs [11]. However, addressing the motivational factors that influence the attitude toward a healthy lifestyle by personalizing interventions to match the needs, motives, and preferences of individuals could result in more engaging and effective health interventions [4,12]. It is well known, for example, from the experiments conducted based on the theories of reasoned action and planned behavior, that the attitude one holds toward a behavior is one of the key determinants for forming the intention to engage in the behavior (or readiness to change the behavior) [13,14].

Values act as guiding principles in life by determining what is important to people [15,16]. According to Schwartz and Bilsky [17], “values (a) are concepts or beliefs, (b) about desirable end states or behaviors, (c) transcend specific situations, (d) guide selection or evaluation of behavior and events, and (e) are ordered by relative importance.” Values are considered as rather stabile motivational characteristics of people, which are related to personality traits [18,19], although changes in value priorities may take place because of changes in life and social conditions [15,18]. As values by definition reflect the motives, needs, and preferences of people, and thereby are one of the factors influencing attitudes [14,20], personalizing eHealth and mHealth interventions according to values may increase the appeal of the interventions and result in higher user engagement. This type of approach has been successfully applied in social marketing, where the message is tailored to the needs and preferences of different target groups [12,21].

To effectively utilize values for personalizing eHealth and mHealth interventions, understanding the relationships between values, well-being, and health behaviors is important. Results of previous studies regarding healthy and unhealthy values in terms of well-being are quite inconsistent (eg, [22-24]), and studies focusing on the relationship between values and health behaviors are sparse. This paper aims to contribute to the knowledge of the associations between values and commitment to values combined with well-being and health behaviors observed in the Finnish population.

### Previous Work

#### Commitment to Values and Well-Being

Previous research indicates that living up to the values one holds important is beneficial for subjective well-being (SWB) [22,25,26]. SWB has been considered as a scientific term for happiness, which comprises 3 primary components—positive affect, negative affect, and life satisfaction [27]. Sharing similar value priorities with one’s social group seems to enhance SWB, as the prevailing environment supports the value-congruent behavior of the person [22,28] and fosters positive interpersonal relationships [29]. Similarly, having values that conflict with social norms may hinder value-congruent behavior [30] and pose a negative influence on SWB [22]. Moreover, people are not always aware of their true, intrinsic value priorities, and differentiating personal values from social expectations may be challenging [26]. Hence, the cognitive process of value clarification and the conscious decision to behave according to or commit to one’s values are sometimes needed for increasing value-congruent behavior and improving well-being [31]. Value clarification and commitment to value-congruent behavior are central concepts in the so-called third wave of cognitive-behavioral therapies [32], which have been effective in treating mental health problems (eg, [33]).

#### Value Types, Well-Being, and Health Behaviors

Schwartz value theory [34] is an extensively studied value classification system, which originally defined 10 broad value types based on the basic human needs, representing different motive orientations. The values form a circumplex structure with 2 axes—openness to change versus conservation and self-transcendence versus self-enhancement. Schwartz value types and the value structure have been recognized and verified in more than 65 different countries. Therefore, the theory is considered as near-universal and applicable across different cultures [34-36]. However, individual differences in the perceived importance attributed to each value type can be substantial [30]. More recently, a version of 11 Schwartz value types has been applied in research, where the Universalism value is divided into 2 subtypes—Nature and Social concern (eg, [37-39]).

A significant amount of research has been focused on the relationships between distinct value types and SWB, (eg, [19,23,24,40,41]). On the basis of the nature of the motivational goals underlying the values, it has been theoretically postulated that values expressing intrinsic goals of autonomy, relatedness, and competence [42] as well as growth needs [18], that is, Self-direction, Stimulation, Universalism, Benevolence, and Achievement, should enhance SWB [22,23]. In contrast, values expressing extrinsic goals such as wealth and fame [42], or deficiency and self-protection needs, that is, Power, Security, Conformity, and Tradition, should have a negative impact on SWB [23,24]. These assumptions were based on early findings, which indicated positive associations of intrinsic goals [43,44] and negative associations of extrinsic goals [43] with SWB.

Recently, Sortheix and Schwartz [24] theorized that values expressing person-focused growth needs (ie, Stimulation, Self-direction, and Hedonism) and the need for relatedness (Benevolence) should be positively associated with SWB. The authors found empirical support for these associations in their large, cross-cultural sample of 32 countries. However, earlier findings regarding the associations between value types and SWB have been quite inconsistent [19,22-24,41]. The most consistent evidence can be found for the negative relationship between valuing Power and SWB [24]. In addition, the observed correlations between the value types and SWB have been mostly weak or moderate [19,22-24,41]. The inconsistent findings could be partly explained by the differences found in socioeconomic and cultural contexts, which can either support or constrain individuals in pursuing their values. For instance, the observed relations of Tradition, Universalism, and Achievement with SWB seem to be opposite in countries with high versus low socioeconomic and egalitarian development [24,45].

The research regarding the associations between value types and health behaviors is sparse and scattered across different behaviors. Most of the studies focus on eating habits (the consumption of fruit and vegetables, calorie-dense food, or meat; and eating out habits) and substance usage (alcohol, tobacco, or drugs). Among Australian participants, Universalism has been observed to be associated with healthy eating habits [46-49], and Hedonism may be associated with overeating [30]. The associations between values and substance usage have been studied particularly among adolescents. One study observed that smoking behavior was related to valuing broadmindedness, independence, and freedom as well as disvaluing obedience [50]. Another study found that extrinsic aspirations (eg, wealth, fame, and public image) were associated with substance use [51]. However, Young and West [52] concluded in their longitudinal study that values may not predict youngsters’ substance use in the long term.

Some studies report a relationship between values and stress-enhancing, exercise, or certain high-risk health behaviors. Valuing health seems to be more related to behaviors that are preventive of direct (eg, drunk driving and smoking) than indirect (eg, seat belt usage and health information seeking) health risks [53]. Furthermore, a study among youngsters found that the (negative) correlations between valuing exciting life and reporting health-risk preventive behaviors were higher than the (positive) correlations with valuing health, whereas for middle-aged adults valuing health was more related to direct health-risk preventive behaviors than valuing exciting life [54]. In eastern and central Europe, risky sexual behavior has been found to have a moderate but consistent relationship with Achievement, Power, Hedonism, Stimulation, and Self-direction [55]. Hedonism may be associated with stress-relieving (relaxing) behavior, whereas Achievement appears to be associated with stress-enhancing behavior (taking on many commitments) [30]. Universalism has been observed to be associated with regular physical activity [47].

Except for the cross-cultural study of Sortheix and Schwartz [24], the reviewed studies regarding values, well-being, and health-related behaviors were relatively small, involving some hundreds of participants. Furthermore, the studies involved mostly younger adults (students) or teachers, thereby limiting the generalizability of the results. Overall, the evidence for associations between values and well-being is still quite inconsistent, and comprehensive research focusing on a multitude of health-related behaviors is lacking.

### This Study

This study aims to discover the associations between self-reported values (commitment to values and value priorities), perceived well-being, and various self-reported health behaviors from a large, cross-sectional dataset of open Web-survey responses, available from more than 100,000 Finnish citizens. The data were collected as part of the Finnish Happiness-Flourishing Study (FHFS), which was a national effort to promote mental well-being and healthy behaviors in the Finnish population [56]. The survey included questions assessing various dimensions of well-being and several different health behaviors. The measures for well-being factors included happiness, depression, life satisfaction, impact of major negative and positive life events on happiness, family- and work-related distress, and communal social activity. The health behavior–related factors comprised exercise, intake of fruits and vegetables, sleep hours, alcohol consumption, and smoking. The data regarding personal values were unstructured including free-text responses.

We adopted an exploratory approach for the data analysis to study whether (1) commitment to values was related to well-being, (2) certain value types could be considered healthier than others in terms of their associations with well-being or health-related behaviors, and (3) previous findings could be replicated with the extensive data at hand. On the basis of previous research, we hypothesized positive associations between well-being and commitment to values [26] as well as between well-being and the value types reflecting intrinsic goals of relatedness and person-focused growth needs [24,43]. Value types reflecting extrinsic aspirations or deficiency needs were expected to be negatively associated with well-being [24]. Associations between value types and health-related behaviors were also expected, especially between Universalism, healthy eating, and regular exercise (eg, [47]).

## Methods

### Study Design

The data were collected at the public website of the FHFS campaign over the period of 1 year, between 2009 and 2010 [56]. FHFS was a national effort to promote mental well-being and a healthy lifestyle in the Finnish population. The study campaign was implemented in collaboration among the National Institute for Health and Welfare, Duodecim Medical Publishing Ltd, a Finnish television (TV) production company (Tarinatalo), and the national public broadcasting company (YLE). The campaign produced a reality TV series about happiness and depression, where celebrities were learning happiness-related skills. The series attracted roughly 250,000 weekly viewers. The FHFS website and the Web survey were part of the campaign (see Multimedia Appendix 1 for the FHFS survey items in Finnish). The FHFS Web survey was advertised during the series episodes and at the website of the broadcasting company. It was freely available to all Finnish-speaking individuals having access to internet. The purpose of the Web survey was to allow participants to measure their happiness levels with the Happiness-Flourishing scale [56] and to encourage them to identify the key sources in life that contributed to their happiness. However, the survey also involved questions about a variety of other well-being factors and health behaviors. On the website of the FHFS survey, it was clearly stated that the collected data would be used for creating public summary reports regarding happiness and the related factors.

The study we conducted was a retrospective and explorative data analysis of the FHFS campaign data, which was driven by our hypotheses regarding the associations between values, well-being, and health behaviors.

### Participants

Altogether, 139,462 anonymous responses were received to the Web survey. The respondents, who did not provide their age or gender, or reported ages below 18 or above 110 years, were excluded from the analyses of discovering associations between variables. In addition, the responses that involved 2 or more unrealistic values for numeric variables were considered unreliable and hence excluded from the study sample. If a response involved an implausible value for 1 numeric variable only, this value was treated as a missing input. The numeric values were considered unrealistic if they did not fall into the following variable-specific ranges—alcohol consumption (0-150 units/week), smoking (0-100 cigarettes/day), weight (30-250 kg), height (70-220 cm), body mass index (BMI, 10-50 kg/m^2^), sleep hours (3-16 hours/day), years of education (from 9 years to the current age of the respondent minus 3, “age-3” years; the compulsory education in Finland takes 9 years), and income (0-5,000,000 Euros/year). After applying these exclusion criteria, 101,130 responses remained in the study sample, of which, 62,625 responses included a list of personal value items. The basic demographics of the study sample are provided in Table 1.

### Materials

#### Well-Being Factors

Perceived happiness was measured using the Happiness-Flourishing scale [56] (Cronbach alpha=.93), which involves 10 items evaluated with a 7-point Likert scale. The score is the sum of the item-specific answers ranging from 10 (*very unhappy*) to 70 (*very happy*). Depression was measured with the Depression Scale [57] (Cronbach alpha=.92). Life satisfaction was assessed by the 7-point Likert-item “How satisfied are you with your life situation right now” (1= *completely unsatisfied* and 7= *completely satisfied*). The validity of employing single-item measures for life satisfaction has been shown in the study by Cheung and Lucas [58].

The impact of major positive and negative life events on happiness was assessed in 3 parts. First, it was enquired whether one had experienced in the past significant negative (eg, divorce, loss of a loved one, prison sentence, unemployment, or serious illness) or positive (eg, new relationship, marriage, retirement, childbirth, new job, or work promotion) changes in life that still mattered. Second, the perceived significance of the reported event was assessed with the item “Estimate the influence of the life event on your happiness nowadays,” having a response scale from 1 (*no influence*) to 10 (*significant negative* or *positive influence*). Finally, the timing of the event was enquired with 5 predefined response options (*within the past 6 months, 1 year, 2 years,* or *5 years,* and *earlier*).

Family- and work-related distress as well as communal social activity were addressed with the following questions: “Do you experience problems in your relationship with your partner?” (problems with partner),“Have your children caused you particular problems?” (problems with children), “How often are you troubled with having to push yourself to the limit in order to cope with your present job or work load?” (work stress), and “How often do you participate in communal social activities or events related to e.g. handicrafts, culture or religion?” (communal social activity). The response options for these questions are presented in the Results section.

**Table 1:** Self-reported demographics of the respondents included in the study sample (N=101,130).

Characteristics		Valid, n (%)^a^	Proportions, %
**Gender**		101,130 (100)	
	Male		21.12
	Female		78.88
**Age (years)**		101,130 (100)	
	18-29		17.12
	30-44		30.37
	45-54		24.60
	55-64		21.22
	≥65		6.70
**Years of education^b^**		86,698 (85.73)	
	<12 (comprehensive school)		13.48
	12-14 (upper secondary education)		27.10
	15-17 (bachelor’s degree or equivalent)		35.04
	>17 (master’s or doctoral degree)		24.39
**Gross household income (Euros/year)**		89,821 (88.82)	
	0-17,999		24.94
	18,000-35,999		24.79
	36,000-59,999		21.12
	≥60,000		29.15
**Body mass index (kg/m^2^)**		99,434 (98.32)	
	<18 (underweight)		1.70
	18-24.99 (normal weight)		50.63
	25-29.99 (overweight)		31.49
	≥30 (obese)		16.19

^a^Proportion of respondents with data available.

^b^The education level (in parenthesis) is estimated based on the Finnish education system.

#### Health-Related Behavior

Physical activity level was assessed with the question “On the average, how much do you exercise or strain yourself physically during your leisure time?” with 4 response options defined by the Gothenburg Scale [59]. According to World Health Organization’s global physical activity recommendations, people should do moderate-intensity activities for at least 2.5 hours per week or vigorous-intensity activities for at least 1 hour and 15 min per week to gain health benefits [60]. Overall, 3 of the 4 response options (performing at least 4 hours of moderate-intensity activities per week, 3 hours of fitness training per week, and athlete training several times a week) indicated of meeting the public health recommendations for physical activity and thus were interpreted as regular exercise and dichotomized into a binary variable.

Healthy eating habits were assessed with the following 2 questions: “On the average, how often do you eat fresh fruits or berries?” and “On the average, how often do you eat fresh vegetables” with 4 response options (*less than once a week, 1-2 times per week, 3-5 times per week, once a day,* and *more often*). According to public health recommendations, people should consume at least 5 portions of fruits and vegetables per day [61]. Thus, the response options of the 2 questions were combined into a binary variable, describing the daily consumption of vegetables, fruits, or berries (healthy eating).

Sleep duration was assessed with the open question “On the average, how many hours do you sleep?” Sleeping 7 to 8 hours per night was regarded as a healthy amount of sleep [62] and dichotomized into a binary variable. Alcohol consumption was assessed with the open question “How many units of alcohol do you drink per week?” accompanied with an explanation for an alcohol unit (1 unit is equivalent to 10-14 g of pure alcohol such as 0.33 L of average-strength beer [4%-7%], 12 cL of wine [10%-15%], or 4 cL of spirits [35%-40%]; [63]). Smoking was assessed with the open question “How many cigarettes, cigars, or pipefuls do you smoke per day?,” and a binary variable was created for representing nonsmoking.

#### Personal Values

The FHFS Web survey was not designed for the purpose of value research; hence, it did not include a validated value survey for assessing personal values. Commonly used tools for value research include the 57-item Schwartz Value Survey (SVS) [34,35] and the Portrait Value Questionnaire (eg, PVQ-21) [64], which define values as “guiding principles in your life” or concepts that are important in one’s life. In the Web survey, the respondents were asked to define the key ingredients of their happiness and were presented with a predefined set of value items via an interactive user interface that allowed to name or choose up to 20 values. A library of more than 200 value items was available in the Web-system, and the respondents could select values from this library as well as freely enter their own items. The predefined value items were presented via a space-like animation, where items from the value library appeared and disappeared in a random order, attempting to resemble twinkling stars in the night sky. The respondents could select values from this value-space by clicking the appearing terms; type words into a search box with predictive text input utilizing the library; or alternatively, enter text from outside the library.

In spite of not employing a traditional value survey, we consider the collected data to represent a good approximation for personal values for the following 2 reasons: (1) one’s “key ingredients of happiness” are most likely personally important concepts in life, just like values are important [15-17]; and (2) exposing the respondents to a predefined library of value items provided a clear clue about the type of data expected from them. Similarly, in the SVS, a list of value items are presented to the respondents [34,35].

Finally, the commitment to live up to one’s personal values (commitment to values) was assessed with the 7-point Likert-item “I have firm values that I strive to nurture” (1= *I totally disagree* and 7= *I totally agree*).

### Statistical Analysis

#### Associations With Commitment to Values

The statistical analyses were performed with the IBM SPSS (version 20) and the free R (version 3.3.1) statistical software. The connections between commitment to values and variables related to well-being factors and health-related behaviors were assessed with multiple linear regression. Visual inspection, pairwise correlations (Pearson and Spearman), descriptive statistics, and principal component analysis (PCA) were used to identify mutually strongly correlated variables among the well-being factors and health behaviors. Depression (*r*_81124_=−.78, *P*<.001) and life satisfaction (*r*_91876_=.72, *P*<.001) correlated strongly with happiness. According to the results of PCA, these variables appeared to align along a common dimension—all had high loadings (.93 for happiness, −.90 for depression, and .87 for life satisfaction) on the same, single component, which explained 80.57% of the overall variability in the data. Variables that did not correlate strongly with each other (| *r* |<.4) were included in the regression model as independent variables. Among the 3 highly correlated variables, only happiness was chosen to be included in the regression model to avoid the problem of multicollinearity. The other variables included were problems with partner, problems with children, work stress, communal social activity, regular exercise, healthy eating, healthy amount of sleep, nonsmoking, alcohol consumption, age, and gender.

One-third of the responses (27,599 out of 82,919), which involved self-assessments regarding commitment to values, had at least 1 of the independent variables missing. Instead of omitting these responses from the regression analysis, multiple imputation (MI) with fully conditional specification (FCS), available in SPSS, was used. MI with FCS is a statistically valid method for creating imputations in large complex datasets that involve both continuous and categorical variables [65]. All the independent variables were included in the imputation model, and 5 sets of imputations were created. For the integer-valued scale variables, the imputed values were rounded. The highest proportion of missing values (20,765/101,130, 20.53%) was imputed for nonsmoking. For most of the other variables, the proportions of missing (imputed) values were less than 5%. The regression analysis was applied on the imputed dataset. The results are presented via the unstandardized beta (*B*) and its 95% CI. Furthermore, squared semipartial correlations (part *r*^*2*^) were calculated separately for each independent variable, adjusted for age and gender, and reported as a measure for the effect size.

The association between commitment to values and the impact of major life events on happiness was assessed separately from the model presented above to involve the timing of the events as a controlling factor. Linear regression was used to study whether commitment to values, controlled for age, gender, and the timing of a major life event, was associated with the impact of the life event. Distinct regression models were built for negative and positive life events. These analyses were performed using the original data, as the impact of major life events was not part of the imputation process. Compared with the other variables of interest, only a small proportion of the responses were related to major life events—altogether, 28,709 and 29,671 responses were included in the analyses regarding negative and positive life events, respectively.

#### Classification of Value Items

The reported value items were classified into value groups based on the Schwartz value theory. Altogether, 779,392 value items described with 23,552 different terms or expressions, including the items with typing errors, were reported in the study sample. Typing errors and infrequent entries were discarded by selecting only those items for classification, which occurred at least 50 times in the data, resulting in 723 different terms.

The classification procedure was conducted in 2 phases. The first phase was performed manually by AH. Obvious synonyms and words, which could be clearly identified to belong under a superordinate category, were renamed with a descriptive common term. For instance, the synonymous words “buddies,” “good friends,” “friendship,” and “friend” were renamed as “friends,” and the words “wife,” “husband,” “spouse,” “boyfriend,” and “girlfriend” were renamed as “partner.” After the renaming procedure, the number of distinctive terms was reduced to 472. This set of terms was then grouped according to the 11 Schwartz value types [37-39] and the related 57-item SVS [34,35]. The words having the same meaning with a Schwartz value item as defined in the SVS were located under the corresponding Schwartz value type. However, many of the reported terms were not represented in the list of Schwarz value items, and language-specific nuances introduced some uncertainty for the matching. Hence, additional non-Schwartz value groups were created for the terms that described similar concepts but could not be matched with any of the Schwartz value items with a complete certainty. Even rather alike concepts were grouped separately to minimize the information loss at this point, despite increasing the likelihood of resulting in highly correlated value groups. As a result, 27 non-Schwartz groups were created in addition to the 11 Schwartz value types.

The second phase of the classification procedure was computational, aiming at investigating whether (1) some value groups correlated strongly with each other and, therefore, could be merged or (2) some value items should be relocated to a different group. The manually classified value items were transformed into a matrix, where the columns represented value types and the rows represented the number of value items each respondent had reported per value type. PCA based on the promax oblique rotation method was used to identify highly correlated dimensions in the value matrix and to verify the appropriate grouping of value items. Only the respondents who had more than 90% of their value items classified with at least 4 classified value items were included in the PCA to diminish the impact of the possible nonsense responses on the classification. The details of the PCA procedure are explained in Multimedia Appendix 2. As a result, the number of non-Schwartz types (groups) was reduced from 27 to 20. Finally, the value types were recoded into binary variables (0=*no items reported* and 1=*at least one item reported* for the value type).

#### Associations With Value Types

Logistic regression was used to study the relationships between the 20 most common value types observed in the study sample and the following well-being and health behavior–related factors: happiness, regular exercise, healthy eating, nonsmoking, and alcohol consumption. Only the respondents who had reported at least 4 value items considered in the value classification were included in the analysis (55,539 out of the 62,625 responses available). This restriction was made to decrease the probability of including nonsense responses that were provided without actual contemplation, for instance, for testing the interactive user interface. Separate logistic regressions were performed for each pair of well-being or health behavior factor and value type, having the binary value type as the dependent. The analyses were adjusted for age and gender. For reference, similar analyses were performed to assess the relationships between the selected well-being or health behavior factors and reporting value items in general (ie, at least 4 classified items) with 92,394 eligible respondents. The results are presented using odds ratios (ORs) with the corresponding *P* values.

## Results

### Statistics of the Responses

A slight majority (52.06%, 43,166/82,919) of the population reported strong commitment to values, and most (63.57%, 64,286/101,130) of the respondents provided a list of their personal value items. A slight majority (51.56%, 48,785/94,617) reported to be happy, though many suffered from work stress and experienced problems with their partners every now and then. Most of the respondents (59.73%, 60,403/101,130) did not share their experiences regarding major negative or positive life events. A clear majority reported healthy behaviors. The descriptive details of the responses are presented in Table 2.

**Table 2:** The self-reported mental well-being and lifestyle characteristics in the study population (N=101,130).

Variable		Valid, n (%)^a^	Proportions, %
**Commitment to values (scale 1-7)**		82,919 (82)	
	Weak (1-3)		7.21
	Moderate (4-5)		40.73
	Strong (6-7)		52.06
**Number of reported value items**		101,130 (100)	
	None		36.43
	1-3		6.03
	4-13		47.62
	14-20		9.91
**Happiness (score 10-70)**		94,617 (93.6)	
	Unhappy (10-30)		5.59
	Neutral (31-50)		42.46
	Happy (51-70)		51.56
**Impact of major negative life events (scale 1-10)**		39,016 (38.58)	
	Weak (1-4)		34.73
	Moderate (5-7)		38.93
	Strong (8-10)		26.34
**Impact of major positive life events (scale 1-10)**		40,727 (40.27)	
	Weak (1-4)		3.94
	Moderate (5-7)		21.24
	Strong (8-10)		74.83
**Problems with partner**		97,809 (96.72)	
	Not in a relationship		26.62
	Never		22.16
	Sometimes		43.87
	Almost all the time		7.35
**Problems with children**		97,903 (96.81)	
	No children		33.56
	Rarely or never		45.52
	Sometimes		14.24
	Almost all the time		6.67
**Work stress**		97,303 (96.22)	
	Not working or studying		13.85
	Rarely or never		24.35
	Sometimes		38.60
	Almost all the time		23.20
**Communal social activity**		98,872 (97.78)	
	At least once a week		27.49
	At least once a month		25.53
	Once or twice a year		22.60
	Rarely or never		24.39


**Regular exercise**		99,580 (98.47)	
	Yes		76.51
	No		23.49
**Daily intake of vegetables, fruits, or berries**		97,621 (96.53)	
	Yes		62.25
	No		37.75
**Sleep 7 to 8 hours**		98,502 (97.40)	
	Yes		74.17
	No		25.83
**Alcohol consumption (units/week)**		92,285 (91.25)	
	0		28.24
	1-5		44.37
	6-10		16.23
	11-16		5.91
	>16		5.26
**Nonsmoker**		80,365 (79.47)	
	Yes		81.47
	No		18.53

^a^The proportion of respondents with data available.

### Associations With Commitment to Values

A significant regression equation was found (*F*_20, 82898_=2123.11, *P*<.001, adjusted *r*^*2*^=0.34) for demonstrating the associations between commitment to values and various well-being and health behavior–related factors. The regression results are presented in Table 3. Among all the variables, happiness showed the strongest (positive) association with commitment to values (part *r*^*2*^=0.28). Involvement in communal social activities (summed part *r*^*2*^=0.09), regular exercise (part *r*^*2*^=0.06), and daily intake of vegetables, fruits, or berries (part *r*^*2*^=0.04) were also positively but weakly associated with commitment to values. Problems with partner, problems with children, work-related stress, healthy amount of sleep, smoking, alcohol consumption, age, and gender were not associated with commitment to values.

Commitment to values was inversely associated with the perceived impact of major negative life events (*B*=−0.35, 95% CI −0.37 to −0.33, part *r*^*2*^=0.03) and positively associated with the perceived impact of major positive life events (*B*=0.28, 95% CI 0.27 to 0.30, part *r*^*2*^=0.04) on happiness, after controlling for age, gender, and the timing of the events. Both regression models were significant (*F*_8,28701_=20427.54, *P*<.001, adjusted *r*^*2*^=0.85 and *F*_8,29663_=97850.55, *P*<.001, adjusted *r*^*2*^=0.96 for negative and positive life events, respectively), though the associations were very weak.

**Table 3:** Linear regression results regarding the associations between commitment to values and various well-being and health behavior–related factors (n=82,919).

Variable		B (95% CI)	Part *r*^2^^a^
Intercept		2.51 (2.46 to 2.57)	—^b^
**Gender (reference=male)**			
	Female	0.11 (0.09 to 0.12)	0.006
Age (years)		0.00 (−0.0 to 0.0)	0.009
Happiness score		0.06 (0.06 to 0.06)	0.281
**Problems with spouse (reference=not in a relationship)**			
	Never	−0.04 (−0.06 to −0.01)	0.014
	Sometimes	−0.07 (−0.09 to −0.05)	0.001
	Always	0.04 (0.01 to 0.07)	0.004
**Problems with children (reference=no children)**			
	Never	0.01 (−0.01 to 0.03)	0.003
	Sometimes	0.00 (−0.02 to 0.03)	0.000
	Always	0.08 (0.04 to 0.11)	0.001
**Work stress (reference=not working or studying)**			
	Never	−0.06 (−0.08 to −0.03)	0.008
	Sometimes	−0.06 (−0.09 to −0.04)	0.002
	Always	−0.02 (−0.05 to 0.01)	0.003
**Communal social activity (reference=less than yearly)**			
	Weekly	0.39 (0.37 to 0.41)	0.050
	Monthly	0.27 (0.25 to 0.29)	0.029
	Yearly	0.15 (0.13 to 0.17)	0.010
**Regular exercise (reference=no)**			
	Yes	0.35 (0.33 to 0.36)	0.055
**Daily intake of vegetables, fruits, or berries (reference=no)**			
	Yes	0.20 (0.18 to 0.22)	0.035
**Sleep 7 to 8 hours (reference=no)**			
	Yes	0.00 (−0.02 to 0.02)	0.009
Alcohol consumption (units/week)		−0.01 (−0.01 to −0.01)	0.010
**Smoking (reference=yes)**			
	No	-0.01 (−0.03 to 0.01)	0.009

^a^Obtained from separate regression models for each variable, adjusted for age and gender.

^b^Not applicable.

### Associations With Value Types

The classified value items covered 94.30% of the 779,392 value-related words or expressions reported. The classification resulted into 11 Schwartz and 20 non-Schwartz value types. However, in this paper, we report results regarding the value types that were expressed at least by 10% of the eligible respondents, that is, all the 11 Schwartz value types and 9 non-Schwartz value types (see Multimedia Appendix 3 for the definitions and exemplary value items for these value types). The 3 most common value types represented in the study sample were the appreciation of Loved ones (non-Schwartz), Hedonism (Schwartz), and Health (non-Schwartz). The most common value type, Loved ones, was reported by 73.13% (40,616/55,539) of the respondents. The prevalence of different value types are provided in Multimedia Appendix 4. The median number of value items classified under the value types was 20 items (range: 1-47 items). Most people used 1 to 2 value items to express a value type, but several value items were also used. For instance, Loved ones could be expressed with a single item “family,” or with several items such as “father,” “mother,” “little sister,” “big brother,” and “child.”

The observed associations between value types and happiness; exercise; intake of vegetables, fruits, or berries; alcohol consumption; and smoking are summarized in Multimedia Appendix 4. The value types having the most significant and extensive associations with happiness and health behaviors, after controlling for age and gender, were Power (social status, dominance—Schwartz), Mental balance (self-acceptance—non-Schwartz), and Health. Smoking; irregular intake of vegetables, fruits, or berries (unhealthy eating); irregular exercise; a 10-unit decrease in the happiness score; and the increase of alcohol consumption by 10 units per week increased the odds of reporting Power values by 27.80%, 27.78%, 24.66%, 20.69%, and 17.35%, respectively. A 10-unit decrease in the happiness score, smoking, unhealthy eating, and irregular exercise increased the likelihood of reporting Mental balance-related values by 24.12%, 20.79%, 16.67%, and 15.37%, respectively. Regular exercise, nonsmoking, and the daily intake of fruits, vegetables, or berries (healthy eating) increased the odds of valuing Health by 71.71%, 39.96%, and 26.76%, respectively.

Other meaningful associations between value types and happiness or certain health behaviors were observed for Tradition (commitment to traditions or religion—Schwartz), Universalism-nature (Schwartz), Stimulation (exciting life—Schwartz), Conformity (with social norms—Schwartz), and the appreciation of Loved ones and Culture (non-Schwartz). The decrease of weekly alcohol consumption by 10 units increased the likelihood of valuing Tradition by 29.30%. Regular exercise increased the odds of reporting Universalism–nature values by 26.09%. Smoking increased the odds of reporting values related to Stimulation and Conformity by 22.62% and 20.48%, respectively, whereas nonsmoking increased the likelihood of valuing Loved ones and naming Culture values by 18.34% and 15.12%, respectively. Unhealthy eating increased the likelihood of reporting Conformity values by 19.46%, whereas healthy eating increased the odds of naming Culture and Universalism–nature values by 15.20% and 13.94%, respectively. A 10-unit increase in the happiness score increased the odds of valuing Loved ones by 17.23%.

A 10-year increase in age increased the odds of naming Conformity values by 29.43%, whereas a 10-year decrease in age increased the odds of valuing Work (non-Schwartz) by 19.12%. Women were more likely to value Home (non-Schwartz), Loved ones, Universalism–nature, Quality of relationships (non-Schwartz), and Health than men with increased odds by 91.19%, 69.73%, 59.74%, 41.06%, and 31.85%, respectively. For men, the odds of reporting Intellectualism (non-Schwartz), Perseverance (non-Schwartz), Conformity, and Achievement (Schwartz) values were increased by 62.52%, 53.54%, 37.80%, and 28.75%, respectively.

In general, women were more likely to report value items than men with the increased odds of 77.08%. There were no major differences observed in the age, happiness, and health-related behaviors between the respondents who reported values and those who did not.

## Discussion

### Principal Findings

We explored whether the self-assessed commitment to one’s values and the reported value items were related to self-reported well-being and health behavior–related factors in a large, cross-sectional sample of Finnish citizens. In our analyses, perceived happiness was considered as the main measure of well-being. As hypothesized, commitment to values was positively, and strongly, associated with happiness. The presumed associations between the value types and happiness were partially supported by our findings. Furthermore, several associations between different value types and health behaviors were observed.

### Comparison With Previous Work

Commitment to values was explored in relation to various well-being and health behavior–related factors. In addition to observing a strong relation between commitment to values and happiness, we discovered that commitment to values was positively associated with frequent communal social activity, regular exercise, and the daily consumption of fruits, vegetables, or berries, though these associations were much weaker compared with happiness. Furthermore, commitment to values seemed to diminish the impact of major negative life events on perceived happiness and strengthen the impact of positive events but with weak associations. Family- and work-related distress, sleep hours, smoking, and alcohol consumption were not associated with commitment to values.

None of the Schwartz values, considered to express the intrinsic aspirations for relatedness and autonomy, or the person-focused growth needs (Stimulation, Self-direction, Hedonism, and Benevolence) were positively associated with happiness, which is somewhat at odds with previous findings [22-24]. However, the appreciation of Loved ones (non-Schwartz value) was positively, although weakly, associated with happiness. We consider valuing Loved ones to express the intrinsic aspiration relatedness—the need to connect with and care for others [42]. Thus, this finding supports earlier observations regarding the positive relation between the aspirations for relatedness and SWB [43,44]. Interestingly, Benevolence was not associated with happiness, though conceptually it may seem similar to Loved ones. Apparently, the motives behind these 2 value types differ somewhat from each other—Valuing Loved ones may reflect both the desire to enhance the welfare of others and the personal need for company, whereas Benevolence values may express mostly the former motive. Hence, valuing Loved ones might express relatedness more fully than Benevolence. However, in the traditional value surveys, these 2 motives are not differentiated from each other.

The Schwartz value Power (social status, wealth, and dominance), considered to express extrinsic aspirations, was negatively associated with happiness, which is consistent with previous findings [24]. In addition, Mental balance (self-acceptance—non-Schwartz) values were negatively associated with happiness. In the study sample, Mental balance values reflected the active process of learning to survive with external pressures, manage stress, and accept one’s incompleteness, which we consider to express deficiency needs. Hence, the finding regarding Mental balance is aligned with the previous results indicating that expressing deficiency needs is negatively associated with SWB [22,24,43].

Our findings confirm many of the previous results regarding the associations between value types and health behaviors but also suggest new, previously unexplored associations. We found that Power, Mental balance, and Health (non-Schwartz) values had the most significant and extensive associations with several health behaviors. Unhealthy behaviors (smoking; insufficient intake of vegetables, fruits, or berries; and irregular exercise) were more prevalent among the respondents who reported Power or Mental balance values compared with those who did not report them. In addition, Power values were associated with slightly increased alcohol consumption. Extrinsic aspirations such as wealth and public image have been previously observed to be related to substance abuse [51]. Furthermore, regular exercise and nonsmoking were considerably more prevalent among respondents who reported Health values compared with those who did not, and healthy eating habits were also related to valuing Health. Likewise, positive associations between valuing Health and reporting healthy behaviors have been observed before [53,54].

In addition, we observed associations between several other value types and selected health behaviors. Reporting Tradition (commitment to traditions or religion—Schwartz) values was associated with decreased alcohol consumption. Conformity (with social norms—Schwartz) and Stimulation (exciting life—Schwartz) values were associated with smoking. The link between smoking and Stimulation values has also been observed before [54]. The appreciation of Universalism–nature (Schwartz) value was associated with regular exercise and healthy eating, though the association with healthy eating was weak. Previously, it has been observed that Universalism values, in general (nature and social concern), are related to healthy habits [46-48]. Our results suggest that this may be true particularly for the nature dimension of Universalism.

Significant gender differences were observed for value priorities. Women especially valued Home (non-Schwartz), Loved ones, and Universalism–nature values, but Quality of relationships (non-Schwartz) and Health values were also important. Men especially valued Intellectualism (non-Schwartz) and Perseverance (non-Schwartz) values, but Conformity and Achievement (Schwartz) values were also common. These results are consistent with the past research on gender differences in personality types (see eg, [66]). At the population level, it has been observed that women score higher in nurturance, gregariousness, and neuroticism traits and seem to be more sensitive to emotions than men. Men tend to be more assertive and intellectually or idea oriented than women. Though these differences have been shown to be pervasive across cultures, they are modest when compared with the individual variation within each gender [66]. Regarding the observed age differences in this sample, Conformity values were more prevalent among older respondents and Work (non-Schwartz) values among younger respondents.

Each of the 11 Schwartz value types were represented in the study sample, but 9 additional value types, reported at least by 10% of the study population (n>5554), were also identified. This finding is unsurprising, as the Schwartz value theory was developed to represent distinctive motive orientations within and across cultures instead of representing all the possible human values [34,35]. Schwartz et al acknowledge that other values do exist, but their meaning may vary considerably between cultures or individuals [36,67]. For instance, valuing health could express either Security (avoiding illness) or Hedonism (enjoying the pleasure of a healthy body) [36].

### Strengths and Limitations

The study is unique in terms of the large sample size and diverse data, including information about various well-being and health behavior–related factors, coupled with personal values. Most of the previous, relevant studies have been restricted regarding the sample size and have involved mostly students or teachers. A welcome exception to these limitations is the recent, large, cross-cultural study of Sortheix and Schwartz [24], which focuses on the associations between value types and SWB. This study covers a broader set of aspects by also including self-reported health behaviors and commitment to values. The age distribution in this sample was representative of the Finnish working-age population at the time of the study. However, the sample is biased toward female respondents and the education level of the respondents was higher than in the general population (Statistics Finland Web database, years 2009-2010 [68]), which is important to keep in mind when considering the generalizability of the results.

We note that the Web survey received responses from people who were attracted by the FHFS campaign, and many of them might have followed some episodes of the happiness-related reality TV series. Thus, especially those people who had a special interest in their well-being, and/or were seeking ways to improve their happiness, might have noticed the survey. Furthermore, those respondents who actively followed the TV series might have already learned some strategies to improve their happiness before answering the Web survey, which could be reflected in their responses, for example, in the value items reported. The social-desirability bias could have also influenced the respondents to evaluate their state of well-being and health behaviors in a more positive light than in reality. However, as this study does not seek to estimate the state of well-being or the value distribution in the population, we consider that the abovementioned matters do not have a significant influence on the results. Although the distributions for happiness, healthy behaviors, and commitment to values were positively skewed, the employed measures captured enough variability to reveal associations between values, happiness, and health behaviors. Furthermore, a variety of value types, covering all the Schwartz value types, was represented in the sample.

We acknowledge that the employed nonvalidated, uncontrolled method for collecting personal values, and assessing commitment to values with a single-item measure could reduce the reliability of the results. However, our study is not the first of a kind to extract knowledge about values from unstructured data and apply the Schwartz value theory in an unconventional setting. Bardi et al [69] measured the national patterns of Americans’ values from newspaper texts by utilizing a value lexicon they derived based on SVS and demonstrated the validity of their approach. Our methods share similarities with their approach, though our study setting was considerably more controlled, as the collected data were closely related to personal values. The single-item measure for commitment to values might have been interpreted slightly differently among the respondents, for example, providing a low score could mean unfamiliarity with the concept of values in general or awareness of one’s values without commitment to them. Nonetheless, the measure was associated positively with the happiness scale, and the observed association was strong.

The major differences between the employed and traditional value surveys are related to the value definition (ie, the question format), survey structure, and the importance ratings of the value items. In the FHFS Web survey, values were defined as the “key ingredients of happiness,” whereas traditionally they are defined as the “guiding principle in your life” or concepts that are important in one’s life [34,35,64]. We suggest that in practice, these definitions are sufficiently similar to each other, as the concepts that produce happiness must also be personally important; therefore, people strive to fulfill them in their choices in life, which is characteristic to values [15-17]. According to the qualitative research of Delle Fave et al [70], the terms used by lay people to describe happiness involve concepts very similar to value items, such as stability, respect to others, just society, harmony, joy, achievement, and autonomy. Moreover, in the Web survey, the respondents were exposed to a predefined library of value items, which provided a clear clue about the type of data that were expected from them. However, responses were not restricted, so people could decide for themselves as to which items were worth reporting. Thus, it is reasonable to assume that the values reported were somehow personally meaningful and hence important.

The value classification scheme was partly subjective, as many of the value items were manually located under Schwartz value types based on the reasoning of one person (AH). However, the exemplary list of value items defined in the 57-item SVS [34,35] was strictly followed; only the items for which obvious, conceptual counterparts could be identified from the SVS were located under Schwarz value types. In addition, PCA was used to verify the hypotheses regarding the appropriate grouping of the remaining ambiguous items.

Despite the abovementioned limitations, the study has the following strengths, which reduce the potential variability and bias in the results. First, we have addressed the main challenges posed by the uncontrolled and unstructured nature of the data in the employed analysis methods. Second, we have a large sample size that is likely to compensate for some of the shortcomings. Conclusions at the population level have been drawn before also from large datasets collected in uncontrolled, scientifically nonvalidated settings, for example, regarding the sleep quality among the users of commercial wearable devices [71]. Third, our interpretations are based on effect sizes rather than on statistical significance in terms of *P* values. Fourth, the resulting value classification is consistent with the results of previous work, as each of the 11 Schwartz values were represented in the study sample. Furthermore, many of the non-Schwartz values, which emerged from our study, are consistent with the classification of Delle Fave et al [70], which is based on qualitative and unstructured data, similar to ours.

### Implications

Understanding the connections between values, well-being, and health-related behaviors could provide valuable insight for the development of engaging eHealth and mHealth interventions that are effective in promoting behavior change and well-being. This large study replicates many of the previous findings related to the associations between value priorities, well-being, and health behaviors and highlights the positive relationship between commitment to values and happiness. In addition, because of the qualitative and unstructured data on values, we found previously unexplored associations—pondering over mental balance issues appeared to be negatively associated with happiness and several health behaviors. Gender differences in reporting values were stark; women emphasized “soft” values (eg, nurture, nature, and health), whereas majority of men reported “hard” values (eg, persistency, achievement, and influence). Valuing Loved ones emerged as a separate value from Benevolence and was associated with happiness, whereas Benevolence was not.

Though this study does not determine causal relations between values and the factors related to well-being and health behaviors, the strong motivational nature of values in guiding attitudes and behaviors, in general, suggests that values could predict behavior, at least via attitudes [17,20,30]. The observed positive association between commitment to values and happiness supports the previously suggested benefits of encouraging value clarification and value-congruent behavior in mental health interventions [31]. Furthermore, knowledge of the associations between values and health behaviors could help identify some of the reasons why one is not motivated to lead a healthy lifestyle, which would enable personalizing interventions to tackle these barriers. People endorsing values that express strong deficiency needs may have more pressing needs to attend before they are able to focus on healthy behaviors. These observed associations between unhealthy behaviors and reporting Mental balance values support this line of thinking. As values reflect the motives, needs, and preferences of people, they could also be utilized for reframing the goals of health behavior change in a more personally appealing way, attempting to create positive personal outcome expectations (ie, behavioral beliefs) associated with healthy behaviors, which in turn would result in a more favorable attitude toward taking action [14]. This type of approach may help engage the unmotivated proportion of the population, not actively interested in health benefits. For example, presenting healthy lifestyle as a means for increasing productivity at work and professional influence might appeal to people valuing Power.

These results along with the motivational nature of values indicate that it is worth to explore how values could be used to personalize and reframe behavior change goals in eHealth and mHealth interventions, and whether this approach would be effective in increasing user engagement at the individual level. The population-level knowledge provided by this study could be utilized in formulating educated hypotheses on how addressing values in eHealth and mHealth interventions may influence user engagement. However, testing these hypotheses would require rigorous research with well-defined, controlled study settings.

Finally, we consider this study as a successful demonstration of the potential of exploiting data collected in uncontrolled settings. Nowadays, the challenge of refining knowledge from unstructured and incomplete data has become ever so relevant, as data from citizens are becoming increasingly available because of the digitalization of societies. This development also provides interesting opportunities for studying the preferences, attitudes, and behavior of citizens.

### Conclusions

This large study suggests that commitment to values is positively associated with happiness and replicates many of the previously observed relationships between value priorities and factors related to well-being and health behaviors. Previously unexplored associations between values, health behaviors, and happiness were also found. Health, Power, and Mental balance values were most relevant in terms of happiness and health behaviors. The results could be utilized in formulating educated hypotheses on how addressing values in eHealth and mHealth interventions may influence user engagement to be tested in controlled study settings.

## Appendix

Multimedia Appendix 1 The Finnish Happiness-Flourishing Study (FHFS) Web Survey.

Multimedia Appendix 2 Details of the principal component analysis (PCA) procedure applied for the classification of value items.

Multimedia Appendix 3 Definitions of the value types expressed by the respondents.

Multimedia Appendix 4 Associations between value types, happiness, and health behavior-related factors.

## Abbreviations

eHealth: electronic health
FCS: fully conditional specification
FHFS: Finnish Happiness-Flourishing Study
mHealth: mobile health
MI: multiple imputation
OR: odds ratio
PCA: principal component analysis
SVS: Schwartz Value Survey
SWB: subjective well-being
TV: television
