# Supplementary material for: Exploring Associations Between the Self-Reported Values, Well-Being, and Health Behaviors of Finnish Citizens: Cross-Sectional Analysis of More Than 100,000 Web-Survey Responses
Source: JMIR Ment Health. 2019 Apr 22;6(4):e12170. doi: 10.2196/12170 (PMC6658231; doi:10.2196/12170)
Supplement: Multimedia Appendix 1 [file mental_v6i4e12170_app1.pdf]

## Appendix 1

## The Finnish Happiness-Flourishing Study (FHFS) Web-Survey

*Happiness-Flourishing Scale:*
01: Kuinka onnellinen olet yleisesti ottaen
1 = En lainkaan onnellinen – 7 = Hyvin onnellinen
02: Kuinka usein olet kokenut onnen hetkiä
1 = En koskaan – 7 = Hyvin usein
03: Kuinka hyvin olet voinut toteuttaa kykyjäsi ja lahjojasi
1 = En lainkaan – 7 = Poikkeuksellisen hyvin
04: Kuinka valoisana näet tulevaisuuden
1 = En lainkaan valoisana – 7 = Hyvin valoisana
05: Kuinka mielekkäänä ja tarkoituksellisena koet elämäsi
1 = En lainkaan mielekkäänä – 7 = Hyvin mielekkäänä
06: Kuinka arvokkaaksi koet elämäsi
1 = En lainkaan arvokkaaksi – 7 = Hyvin arvokkaaksi
07: Kuinka hyvin koet hallitsevasi elämääsi
1 = Heikosti – 7 = Erittäin hyvin
08: Koetko olevasi onnellisempi kuin muut tuntemasi ihmiset
1= En lainkaan onnellisempi – 7 = Erittäin paljon onnellisempi
09: Missä määrin koet, että sinulla on annettavaa muille ihmisille
1= Ei lainkaan annettavaa – 7 = Erittäin paljon annettavaa
10: Missä määrin olet saavuttanut sisäisen rauhan
1 = En lainkaan – 7 = Täydellisesti

*Life satisfaction related questions:*

et01: Kuinka tyytyväinen olet läheisiin ihmissuhteisiisi juuri nyt?
et02: Kuinka tyytyväinen olet taloudelliseen tilanteeseesi juuri nyt?
et03: Kuinka tyytyväinen olet elämän olosuhteisiisi yleensä juuri nyt?
et04: Kuinka tyytyväinen olet omaan terveyteesi yleensä juuri nyt?
et05: Kuinka tyytyväinen olet läheisesi terveyteen yleensä juuri nyt?

et01-et05
1 = Erittäin tyytymätön – 7 = Erittäin tyytyväinen

et06: Kuinka kovaa tai helppoa elämäsi on juuri nyt?
1 = Erittäin kovaa – 7 = Erittäin helppoa
et07: Kuinka ikävää tai iloista elämäsi on juuri nyt?
1 = Erittäin ikävää – 7 = Erittäin iloista

*Depression Scale (DEPS)*
dep01: Olen kärsinyt unettomuudesta
dep02: Olen tuntenut itseni surumieliseksi
dep03: Minusta on tuntunut, että kaikki vaatii ponnistusta
dep04: Olen tuntenut itseni tarmottomaksi
dep05: Olen tuntenut itseni yksinäiseksi
dep06: Tulevaisuus on tuntunut toivottomalta
dep07: Elämästä nauttiminen on tuntunut mahdottomalta
dep08: Olen tuntenut itseni arvottomaksi
dep09: On tuntunut, että kaikki ilo on hävinnyt elämästä
dep10: On tuntunut, että alakuloisuuteni ei ole hellittänyt edes perheeni tai ystävieni avulla

dep01–dep10
1 = Erittäin paljon – 4 = Ei lainkaan

*Happiness skills related questions*
ot01: Omistan suuren osan ajastani lähimmäisilleni
ot02: Olen hyvin kiitollinen kaikesta saamastani ja saavuttamastani
ot03: Muiden ihmisten auttaminen on minulle ominaista
ot04: Suhtaudun tulevaisuuteen hyvin luottavaisesti
ot05: Haluan elää juuri tätä hetkeä
ot06: Ulkoilen, liikun tai urheilen säännöllisesti
ot07: Minulla on pysyviä arvoja, joita pyrin edistämään
ot08: Koen vastoinkäymiset haasteina
ot09: Uppoudun usein työhöni ja askareisiini niin, että aika unohtuu
ot10: Anteeksiantaminen on minulle helppoa
ot11: Saan voimaa hiljentymisestä ja mietiskelystä
ot12: Minulla on selkeitä tavoitteita elämässäni

ot01–ot10
1 = Täysin eri mieltä – 7 = Samaa mieltä

*Gender:* 1 = nainen, 2 = mies

*Age:* Minkä ikäinen olet

*Height:* Kuinka pitkä olet (cm)

*Weight:* Kuinka paljon painat (kg)

*Postal code:* Postinumerosi kaksi ensimmäistä numeroa

*Income:* Kuinka suuret olivat taloutesi kokonaistulot viime vuonna bruttona (euro)

*Household size related questions*
01: Taloudessasi asuu, itsesi mukaan lukien ___ aikuista
02: Taloudessasi asuu, itsesi mukaan lukien ___ lasta

*Years of education:* Kuinka monta vuotta olet yhteensä käynyt koulua tai opiskellut päätoimisesti

*Exercise:* Kuinka paljon keskimäärin liikut ja rasitat itseäsi ruumiillisesti vapaa-aikana
1 = Vapaa-aikanani en paljonkaan liiku
2 = Vapaa-aikanani kävelen, pyöräilen tai liikun muulla tavalla vähintään 4 tuntia viikossa
3 = Harrastan vapaa-aikanani varsinaista kuntoliikuntaa vähintään 3 tuntia viikossa.
4 = Harjoittelen vapaa-aikanani kilpailumielessä säännöllisesti useita kertoja viikossa rasittavia urheilumuotoja.

*Financial situation:* Onko taloudellinen tilanteesi nyt parempi vai huonompi kuin aikaisemmin
1 = paljon parempi
2 = hieman parempi
3 = suunnilleen samanlainen
4 = hieman huonompi
5 = paljon huonompi

*Problems with spouse:* Onko sinulla vaikeuksia tulla toimeen puolisosi kanssa
1 = En ole parisuhteessa
2 = Melkein koko ajan
3 = Joskus
4 = Ei koskaan

*Problems with children:* Onko lapsista aiheutunut sinulle erityisiä vaikeuksia
1 = Minulla ei ole lapsia
2 = Melkein koko ajan
3 = Aika usein
4 = Joskus
5 = Harvoin
6 = Ei koskaan

*Vegetables consumption:* Kuinka usein keskimäärin syöt tuoreita vihanneksia tai juureksia
1 = Harvemmin kuin kerran viikossa
2 = 1-2 päivänä viikossa
3 = 3-5 päivänä viikossa
4 = Päivittäin

*Fruits and berries consumption:* Kuinka usein keskimäärin syöt tuoreita hedelmiä tai marjoja
1 = Harvemmin kuin kerran viikossa
2 = 1-2 päivänä viikossa
3 = 3-5 päivänä viikossa
4 = Päivittäin

*Work stress:* Miten usein sinua kiusaa se, että joudut pinnistämään voimasi äärimmilleen pystyäksesi selviytymään nykyisestä työstäsi tao työmäärästäsi
1 = En ole ansiotyössä, enkä opiskele päätoimisesti
2 = Melkein koko ajan
3 = Aika usein
4 = Joskus
5 = Harvoin tai ei koskaan

*Communal social activity:* Kuinka usein osallistut seura- tai yhdistystoimintaan, harrastat esim. kädentaitoja tai kulttuuria tai käyt uskonnollisissa tilaisuuksissa
1 = Kerran viikossa tai useammin
2 = Kerran kuukaudessa tai useammin
3 = Kerran tai muutaman kerran vuodessa
4 = Harvemmin tai en koskaan

*Binge drinking:* Kuinka usein juot alkoholijuomia niin, että tunnet itsesi päihtyneeksi
1 = Ainakin pari kertaa viikossa
2 = Ainakin kerran viikossa
3 = Ainakin kerran kuukaudessa
4 = Harvemmin kuin kerran kuukaudessa

*Alcohol consumption*: Kuinka monta annosta alkoholia juot viikossa

*Smoking:* Kuinka monta savuketta, sikaria tai piipullista poltat päivittäin

*Total cholesterol:* Mikä oli viimeisin kolesteroliarvosi, kokonaiskolesteroli (10* mmol/l, esim 57 = 5,7 mmol/l)

*HDL cholesterol:* Mikä oli viimeisin kolesteroliarvosi, HDL-kolesteroli (10* mmol/l, esim 14 = 1,4 mmol/l)

*Sleep hours:* Kuinka monta tuntia nukut keskimäärin

*Questions about major negative life events:*

01: Onko sinulle tapahtunut jokin huomattava kielteinen tai rankka elämänmuutos joka vaikuttaa yhä

1 = Asumusero
2 = Avioero
3 = Lapsen kuolema
4 = Läheisen perheenjäsenen kuolema
5 = Ystävän kuolema
6 = Puolison kuolema
7 = Vankilatuomio
8 = Työstä erottaminen
9 = Loukkaantuminen
10 = Vakava sairastuminen
11 = Perheenjäsenen sairastuminen
12 = Muu, mikä?
13 = En tahdo kertoa

02: Milloin
1 = Puolen vuoden sisällä
2 = Vuoden sisällä
3 = 2 vuoden sisällä
4 = 5 vuoden sisällä
5 = Aikaisemmin

03: Arvioi elämänmuutoksen vaikutusta onnellisuuteesi nyt
1 = Ei vaikutusta – 10 = Suuri kielteinen vaikutus

*Questions about major positive life events:*

01: Onko sinulle tapahtunut jokin huomattava myönteinen elämänmuutos joka vaikuttaa yhä
1 = Uusi ihmissuhde
2 = Avioliitto
3 = Eläkkeelle siirtyminen
4 = Lapsen syntymä
5 = Lapsenlapsen syntymä
6 = Muutto uuteen kotiin
7 = Opiskelupaikka
8 = Työpaikka
9 = Ylennys
10 = Palkankorotus
11 = Muu, mikä
12 = En tahdo kertoa

02: Milloin
1 = Puolen vuoden sisällä
2 = Vuoden sisällä

3 = 2 vuoden sisällä
4 = 5 vuoden sisällä
5 = Aikaisemmin

03: Arvioi elämänmuutoksen vaikutusta onnellisuuteesi nyt
1 = Ei vaikutusta – 10 = Suuri kielteinen vaikutus
