# Supplementary material for: Exploring Associations Between the Self-Reported Values, Well-Being, and Health Behaviors of Finnish Citizens: Cross-Sectional Analysis of More Than 100,000 Web-Survey Responses
Source: JMIR Ment Health. 2019 Apr 22;6(4):e12170. doi: 10.2196/12170 (PMC6658231; doi:10.2196/12170)
Supplement: Multimedia Appendix 2 [file mental_v6i4e12170_app2.pdf]

Appendix 2

Details of the PCA Procedure Applied for the Classification of Value Items

Principal component analysis (PCA) based on the promax oblique rotation method was applied to investigate whether 1) certain non-Schwartz value groups could be merged with other value groups, or 2) some value items should be relocated to a different group. Before performing the analysis, the data were transformed into a value matrix, where columns represented value types and rows represented the number of value items each respondent had reported per value type. PCA was applied in numerous settings and the value matrix was accordingly, iteratively modified via the following steps: Each of the non-Schwartz value groups were explored one by one to verify whether their value items were adequately located by 1) dividing the non-Schwartz value group into subsets of items (usually 1-3 words) that strictly represented a single concept, 2) creating temporary value groups to the value matrix for each subset, 3) applying PCA to the modified value matrix, 4) merging the value groups in the matrix according to the PCA results, and 5) repeating the steps 3 and 4 until all the temporary value groups were merged with other groups, or no further merges were suggested by the PCA.

The number of components in the each PCA solution was defined based on the eigenvalue (0.98 – 1.0), scree plot, and the conceptual sensibility of the components. Only the value groups with communalities and maximum component loadings ≥ 0.3 were included in the final PCA solutions. Value groups were merged, if they were univocally loaded on the same component with loadings ≥ 0.4 amongst non-Schwartz value items (between temporary groups, or temporary groups and non-Schwartz groups), or with loadings ≥ 0.6 when Schwartz groups were involved (between temporary groups and Schwartz value types), and if the groups seemed conceptually compatible. These thresholds were defined based on the commonly observed loadings across different PCA settings and the conceptual sensibility of the components associated with varying loading magnitudes.

Altogether, PCA was applied in 33 different settings, where the non-Schwartz value group under investigation and the value matrix with the corresponding temporary value groups varied. The number of components in the PCA solutions varied from 7 to 16. The components of the different PCA solutions explained from 41% to 67 % of the overall variability in the value groups.
