# Supplementary material for: Exploring Associations Between the Self-Reported Values, Well-Being, and Health Behaviors of Finnish Citizens: Cross-Sectional Analysis of More Than 100,000 Web-Survey Responses
Source: JMIR Ment Health. 2019 Apr 22;6(4):e12170. doi: 10.2196/12170 (PMC6658231; doi:10.2196/12170)
Supplement: Multimedia Appendix 3 [file mental_v6i4e12170_app3.pdf]

Appendix 3

Definitions of the value types expressed at least by 10% of the respondents (n=55,539[a]).

Value type	Conceptual definition	Exemplary value items
Power (S)	Social status and prestige, control or dominance over people and resources, *outward appearance*	Social power, authority, wealth, preserving public image, *elegance, attractiveness*
Achievement (S)	Personal success through demonstrating competence according to social standards, *skillful at solving problems, good standard of living*	Ambitious, successful, capable, influential, *expertise, analytical, pleasant housing conditions*
Hedonism (S)	Pleasure or sensuous gratification for oneself, *sentimentality, sense of humor*	Pleasure, enjoying life, self-indulgent, *intimacy, day-dreaming, playfulness, laughter*
Stimulation (S)	Excitement, novelty, and challenge in life	Daring, a varied life, an exciting life
Self-direction (S)	Independent thought and action – choosing, creating, exploring; *devotion to personally meaningful activities*	Creativity, freedom, independent, choosing own goals, curious, *self-fulfillment, inspiration*
Universalism – Nature (S)	Appreciation and protection for the nature, *enjoying the nature*	Protecting the environment, unity with nature, a world of beauty, *art, spending time in the nature, outing*
Universalism – Social concern (S)	Understanding, appreciation, tolerance and protection for the welfare of all people	Equality, social justice, wisdom, broadminded, a world at peace, *kindness, communication skills, rectitude*
Benevolence (S)	Preservation and enhancement of the welfare of people with whom one is in frequent personal contact	Helpful, honest, forgiving, loyal, responsible, *generous, empathic, unselfish*
Tradition (S)	Respect, commitment, and acceptance of the customs and ideas that traditional culture or religion provide	Devout, respect for tradition, humble, moderate, accepting my portion in life, *patriotism, hope, thankfulness*

Conformity (S)	Restraint of actions, inclinations, and impulses likely to upset or harm others and violate social expectations or norms	Self-discipline, politeness, honoring of parents and elders, obedient
Security (S)	Safety, harmony, and stability of society, of relationships, and of self	Family security, national security, social order, clean, reciprocation of favors, *piece of mind, financial security, accurateness, cautiousness, sensibility, patience*
Loved ones	Important people with whom one is in frequent personal contact	Partner, family, friends, siblings, grandchildren
Health	Health-conscious, interest towards a healthy lifestyle	Health, nutrition, fitness, healthcare
Mental balance	Knowing the self, accepting and appreciating the person one has become, nurturing mental well-being, defeating personal obstacles	Self-respect, spiritual growth, stress management, survival, being in control of one's life, peacefulness
Quality of relationships	Cultivation of relationships, interest to improve the quality of one's interpersonal relations	Companionship with partner, parenting, friendship, love, trust, respect
Culture	Attending cultural events, enjoying entertainment, producing art and handicrafts	Theatre, literature, travelling, music, dance, movies, creating artwork, handcrafting
Perseverance	Firm attitude in handling affairs, active and influential member of the society and communities	Persistency, fortitude, toughness, concentrating on the essential in problem solving, politics, community activities, desire to be heard and seen
Work	Special interest in work and studies	Occupation, workplace, studies
Home	Appreciation for home	Home
Intellectualism	Appreciation for intellectuality, idealism, and education; demand for progress and development in the society and the world	Spirituality, meditation, philosophy of life, education, internationality, public services

Notes. Schwartz values are denoted with (S).The definitions and value items for Schwartz values are adapted from [36] and completed with additional value dimensions and items (*in italics*) that were observed to reflect them in the present study.

[a]Includes the respondents, who reported at least four classified value items.
