# Supplementary material for: Exploring Associations Between the Self-Reported Values, Well-Being, and Health Behaviors of Finnish Citizens: Cross-Sectional Analysis of More Than 100,000 Web-Survey Responses
Source: JMIR Ment Health. 2019 Apr 22;6(4):e12170. doi: 10.2196/12170 (PMC6658231; doi:10.2196/12170)
Supplement: Multimedia Appendix 4 [file mental_v6i4e12170_app4.pdf]

Appendix 4

Odds ratios (OR) regarding the associations between value types, happiness, and health behavior-related factors (n=55,539)[a].

Value type	Preval. (%)	Age (OR / 10 years)	Female (OR)	Happiness score (OR / 10 units)	Alcohol (OR / 10 units / week)	Regular exercise (OR)	Healthy eating[b] (OR)	Non-smoking (OR)
**Loved ones**	73.13	0.93***	**1.70*****	**1.17*****	0.96**	1.08**	1.10***	**1.18*****
**Hedonism (S)**	67.65	0.94***	0.91***	1.02*	1.07***	1.03	1.00	0.93**
**Health**	54.97	**1.12*****	**1.32*****	1.07***	0.93***	**1.72*****	**1.27*****	**1.40*****
**Mental balance**	52.78	1.04***	**1.17*****	**0.81*****	1.03*	**0.87*****	**0.86*****	**0.83*****
**Universalism - concern and tolerance (S)**	48.66	**1.12*****	0.92***	0.99	0.98	**0.90*****	0.94**	**0.86*****
**Benevolence (S)**	47.57	**1.19*****	**1.11*****	1.04***	0.93***	0.95*	**0.89*****	**0.85*****
**Universalism – nature (S)**	42.36	**1.13*****	**1.60*****	0.99	1.01	**1.26*****	**1.14*****	**1.12*****
**Self-direction (S)**	39.66	**0.90*****	0.92***	0.99	1.01	0.98	1.08***	0.99
**Achievement (S)**	38.55	**1.14*****	**0.78*****	0.91***	1.01*	0.91***	**0.91*****	**0.88*****
**Quality of relationships**	34.25	0.94***	**1.41*****	1.03***	1.00	1.00	0.98	0.94*
**Security (S)**	34.01	1.08***	0.92***	0.91***	0.98	**0.90*****	**0.88*****	0.92**
**Culture**	29.24	1.07***	**1.12*****	0.99	1.07***	0.98	**1.15*****	**1.15*****
**Tradition (S)**	25.61	**1.15*****	1.09**	1.05***	**0.77*****	0.96	0.97	**1.12*****
**Power (S)**	16.59	**0.90*****	0.92**	**0.83*****	**1.17*****	**0.80*****	**0.78*****	**0.78*****
**Perseverance**	16.39	0.98**	**0.65*****	0.95***	0.99	0.94*	0.92***	**0.87*****
**Stimulation (S)**	16.03	1.00	0.96	1.03*	1.03	1.05	0.97	**0.82*****
**Work**	15.92	**0.84*****	1.10**	**1.13*****	1.00	1.03	1.01	1.00
**Home**	14.77	1.02*	**1.91*****	**1.12*****	0.95*	0.95	1.02	1.14**
**Intellectualism**	13.31	**1.11*****	**0.62*****	1.01***	1.02***	0.91**	1.02*	**0.84*****
**Conformity (S)**	12.27	**1.29*****	**0.73*****	0.93***	0.96*	**0.89*****	**0.84*****	**0.83*****
**Any values[c]**	60.11	0.92***	**1.77*****	1.07***	0.92***	1.01	1.04*	1.05*

[a]The odds ratios are bolded for which the change in odds per unit is at least 10% and *P*<.001. Schwartz value types are denoted with (S).

[b]Daily intake of vegetables, fruits or berries.
[c]The respondents with at least 4 classified value items are compared with those who did not report any value items (n=92,394).
**P*<.05, ***P*<.01, ****P*<.001.
